# Sex-Related Expression of Klotho in Rat Kidneys: Species Differences Between Rats and Mice

**DOI:** 10.3390/ijms27020716

**Published:** 2026-01-10

**Authors:** Davorka Breljak, Dean Karaica, Ivana Vrhovac Madunić, Vedran Micek, Tatjana Orct, Marija Ljubojević, Dubravka Rašić, Željka Vogrinc, Saša Kralik, Marko Gerić, Goran Gajski, Ivana Novak Jovanović, Lucia Nanić, Jasna Jurasović, Maja Peraica, Ivica Rubelj, Ivan Sabolić

**Affiliations:** 1 Division of Toxicology, Institute for Medical Research and Occupational Health, 10 000 Zagreb, Croatia; 2 Laboratory Animals Unit, Institute for Medical Research and Occupational Health, 10 000 Zagreb, Croatia; 3 Division of Occupational and Environmental Health, Institute for Medical Research and Occupational Health, 10 000 Zagreb, Croatia; 4 Department of Laboratory Diagnostics, University Hospital Centre, 10 000 Zagreb, Croatia; 5 Laboratory for Molecular and Cellular Biology, Division of Molecular Biology, Ruđer Bošković Institute, 10 000 Zagreb, Croatia

**Keywords:** distal tubules, kidney, *kl*/*kl* mice, inorganic phosphate, membrane-bound Kl, proximal tubules, sexual dimorphism

## Abstract

The anti-aging gene/protein Klotho (Kl), most present in kidneys, has been well studied in mice (mKl), but not in rats (rKl). This study investigated the renal rKl expression in male and female rats. Sex-related measurement of rKl-controlled electrolytes was performed in plasma/urine samples, as were tests on species differences in renal Kl expression (rats vs. mice). rKl mRNA/protein expression was studied by qRT-PCR/Western-blotting in renal total RNA/cell membranes and its localization by immunofluorescence microscopy. Urine/plasma ions (phosphate/total calcium) and macroelements (phosphorus/calcium) were measured biochemically and by ICP-MS, respectively. In rat kidneys, the rKl mRNA/protein was detected in the cortex, outer and inner stripe but not in the papilla, and was immunolocalized in the basolateral membrane of proximal tubules in the cortex and outer stripe, but not in the intercalating cells of the cortical distal tubules, whereas mKl was observed in the mouse kidney cortex but not the outer stripe. Female-dominant expression of renal rKl, affected by androgen’s inhibitory effect, may have contributed to the sex-related level of urine electrolytes, particularly phosphates. Renal mKl expression was male-dominant. Sex- and species-related differences in renal Kl expression may be relevant for the selection of the sex and/or the model organism in studies addressing aging/mineral homeostasis.

## 1. Introduction

The α-Klotho, referred to as Klotho (symbol *Kl* and *KL* for rodents and humans, respectively), is an anti-aging gene whose disruption shortened the lifespan of *Kl*-hypomorphic (*kl*/*kl*) as well as *Kl*-knockout (*kl^−^*^/*−*^) mice by inducing the premature-aging syndrome in comparison to wild-type (WT) (*kl*^*+*/*+*^) mice [1,2]. Conversely, the overexpression of the *Kl* gene extended the lifespan and ameliorated aging and age-related syndrome in mice, revealing *Kl* as a senescence-related gene [2].

In mammals, *KL*/*Kl* mRNA is expressed in various organs and tissues, with the highest expression in kidneys, encoding the highly conserved ~130 kDa single-pass membrane protein composed of the short intracellular domain, large extracellular domain, and transmembrane domain, thus forming the membrane-bound form (Kl^TM^), highly expressed in the renal tubular epithelium [1,3,4,5]. Kl also exists as a soluble protein (Kl^ecto^) composed of either full-length or two smaller extracellular fragments, being cleaved *via* ectodomain shedding and detected in extracellular fluids such as blood, proximal tubule luminal fluid, urine, and cerebrospinal fluid [6,7,8].

Both Kl^TM^ and Kl^ecto^ regulate the level of inorganic phosphate (Pi), calcium ions (Ca^2+^), and vitamin D3 (calcitriol) *via* three mechanisms: (1) by suppressing the renal Pi reabsorption, thus promoting the phosphate excretion into urine, (2) by promoting the renal Ca^2+^ reabsorption, thus preventing its loss in urine, and (3) by inhibiting the renal calcitriol synthesis, thus maintaining the mineral ion homeostasis [2,9,10,11]. The membrane Kl^TM^ binds to fibroblast growth factor receptors (FGFRs) and functions as an obligatory coreceptor for fibroblast growth factor 23 (FGF23), thus driving the FGF23 signal pathways in *Kl*-positive tissues, whereas the circulating Kl^ecto^ acts as a scaffold protein for FGF23, thus promoting the FGF23 signaling in both *Kl*-positive and *Kl*-negative tissues [9,11,12,13].

In rat and mouse kidneys, Kl^TM^ was found to be predominantly expressed in the basolateral membrane (BLM) of distal nephron segments including distal convoluted tubules (DCT), connecting tubules (CNT), and cortical collecting ducts (CCD), and to a lesser extent, in the BLM of proximal tubules (PT), including proximal convoluted tubules (PCT), but not proximal straight tubules (PST) [1,5,6,14,15,16,17]. In contrast to PCT, which are lined by the same type of epithelial cells, the epithelia of DCT, CNT, and CCD in rodent kidneys are lined by morphologically distinct cells; distal and connecting tubule cells are the major cell type in DCT and CNT, respectively, whereas principal cells gradually replace the CNT cells and represent the major cell type in CCD, while intercalated cells are scattered between them in CNT and CCD [18,19,20]. However, the distribution of Kl-positive cells lining the distal nephron tubules (CNT and CCD) has not been detailed so far in mouse and rat kidneys, especially in terms of species differences (mice vs. rats), possibly because some differences have not been suspected due to the high homology of their Kl cDNA/protein [1,4,21].

In recent years, the National Institutes of Health has emphasized the importance of sex as a biological variable (SABV) in human and animal studies, as well as highlighted the consideration of both sexes in experimental design and data dissemination [22,23,24]. Furthermore, the impact of sex hormones on the mammalian renal transportome has been extensively documented [25,26,27,28,29]. The regulating role of testosterone on renal mKl expression in mice has been shown [30], but relevant sex-related data are still missing in rats as a frequently used animal model.

Here, we tested commercial anti-Kl antibodies (Kl-Ab) as a crucial tool for Western blotting (WB) and immunofluorescence cytochemistry (IFC), and investigated the zonal expression of rKl along the nephron at both mRNA and protein. Furthermore, we investigated the sex-related expression of rKl in the kidneys of male and female Wistar rats, and the effect of sex hormones on its mRNA and protein expression following a gonadectomy. Finally, sex-related measurements of Pi, total calcium (tCa), essential macroelements phosphorus (P), and calcium (Ca) were performed in the plasma and urine samples of sham-operated and gonadectomised male and female rats. Species differences between rKl and mKl in renal Kl immunodistribution, as well as their sex-related expression, were also considered.

Knowledge of the sex-related expression of renal rKl is important for a deeper understanding of the sex-related differences in mineral homeostasis, as well as aging and longevity in rats, as a frequently used model in today’s research. Since kidneys represent the principal source of both Kl^TM^ and Kl^ecto^, sex-related (male vs. female) and species-related (rat vs. mouse) differences in renal Kl expression could be relevant for the optimal selection of sex and/or animal models in further basic/preclinical studies addressing the aging, longevity, and (patho)physiology of mineral homeostasis.

## 2. Results

### 2.1. Testing of Kl-Abs

**Figure 1:**
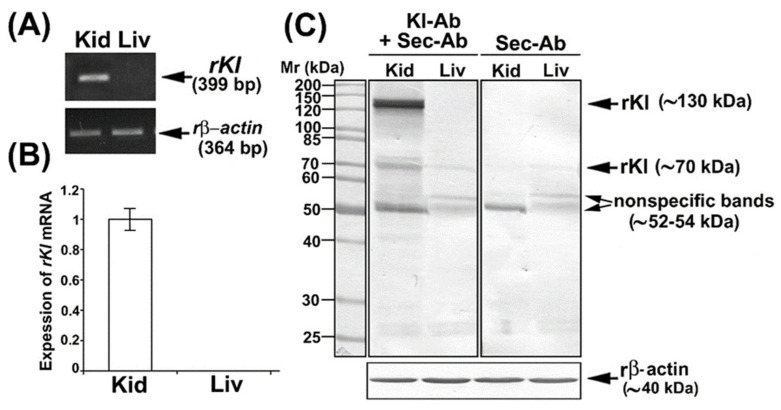
Expression of *rKl* mRNA in rat tissues and characterization of Kl-Ab (KO6039) by Western blotting. (**A**) Expression of *rKl* mRNA in rat kidney and liver was studied by end-point RT-PCR using the housekeeping gene *rβ-actin* as an endogenous control. The figure shows images of agarose gel electrophoresis representative of similar findings in cDNA preparations from three male rats. (**B**) Expression of *rKl* mRNA in rat kidney and liver was measured by qRT-PCR and analyzed by comparative 2^−ddCt^ method using the *rβ-actin* as endogenous control. *rKl* mRNA expression in the liver was plotted relative to the *rKl* mRNA in the kidney; also shown is the mean ± SEM of data collected from cDNA preparations of three male rats. (**C**) Western blot of TCM proteins from rat kidney and liver was studied with the Kl-Ab (KO603) using the rβ-actin as a loading control. Relative molecular mass (Mr, kDa) of protein bands was estimated by the protein ladder. Each well contained 80 μg protein. The figure also shows images of Western blotting representative of similar findings in TCM preparations from three male rats. Kid = kidney; Kl-Ab = anti-Klotho antibody; Liv = Liver; Sec-Ab = secondary antibody; Mr = relative molecular mass.

We first tested *rKl* mRNA expression in the rat kidneys and liver by RT-PCR in order to define optimal tissue controls as shown in Figure 1A; the *rKl*-related RT-PCR product 399 bp was detected in rat kidneys but not livers, whereas expression of housekeeping gene *rβ-actin* (364 bp) was similar in both organs, demonstrating thus an equal cDNA input. The same *rKl* mRNA pattern was observed by qRT-PCR as illustrated in Figure 1B, confirming thus the kidney and liver as *rKl*-positive and *rKl*-negative tissues, respectively. We further tested the commercially available Kl-Abs by WB and IFC.

In WB studies, we used the Kl-Ab (KO603; Clone No. KM2076) that has been previously validated in *Kl*-hypomorphic mice, but has not been tested in total cell membranes (TCMs) isolated from rat kidneys [5]. To check for a possible non-specific binding of the secondary antibody (Sec-Ab), the renal and hepatic TCM samples were additionally incubated with Sec-Ab only. In TCM isolated from the *rKl*-positive rat kidney, Kl-Ab + Sec-Ab labeled three protein bands (~130, ~70, and ~52–54 kDa) (Figure 1C; Kl-Ab + Sec-Ab; Kid), whereas ~130 and ~70 kDa bands were not detected when the Sec-Ab was applied alone (Figure 1C; Sec-Ab; Kid). In TCM isolated from the *rKl*-negative liver, where the rKl protein expression was not expected, Kl-Ab + Sec-Ab labeled two bands (~70 and ~52–54 kDa) (Figure 1C; Kl-Ab + Sec-Ab; Liv) that were also present with Sec-Ab only (Figure 1C; Sec-Ab; Liv). Keeping in mind the molecular mass of detected bands as well as the ability of this antibody (KO603) to recognize both rKl forms, the strongly stained band of ~130 kDa was assumed to represent a full-length rKl^TM^, whereas the band of ~70 kDa could represent rKl^ecto^. By additional WB studies, we could not detect either entire or smaller fragments of soluble rKl^ecto^ in rat plasma and urine. The bands of ~52–54 kDa in the renal and hepatic TCM were estimated as non-rKl-related due to non-specific Sec-Ab binding. In further comparative studies, we have quantified only the full-length rKl~-band of ~130 kDa.

**Figure 2:**
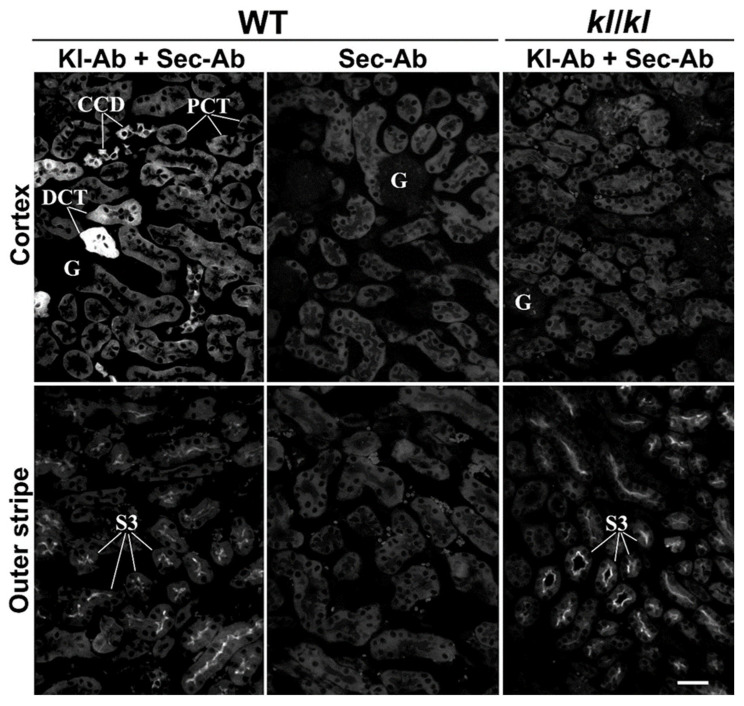
Characterization of Kl-Ab (AF1819) by immunofluorescence cytochemistry. The figure shows images of immunofluorescence staining with Kl-Ab (AF1819) representative of similar findings in cryosections from three wild-type (kl^+/+^) and three Klotho-deficient (*kl*/*kl*) male mice. Individual tubules were identified based on their morphology and localization in the specific nephron zones, including cortex (proximal convoluted tubules, distal convoluted tubules, and cortical collecting ducts) and outer stripe (proximal straight tubules). The scale bar is 20 μm. CCD = cortical collecting ducts; DCT = distal convoluted tubules; G = glomerulus; *kl*/*kl* = klotho-hypomorphic mice; Kl-Ab = anti-Klotho antibody; PCT = proximal convoluted tubules; Sec-Ab = secondary antibody; S3 = proximal straight tubules; WT = wild-type.

Using the same Kl-Ab (KO6039) in IFC experiments, we could not detect any rKl-related staining in the cryosections of rat kidneys, although we applied multitudes of antigen-retrieval protocols [31,32]. Therefore, we tested another commercial Kl-Ab (AF1819) that had been previously used for Kl detection in rodent brains [33]. Following optimal antigen retrieval, we first compared renal sections of WT and *Kl*-hypomorphic (*kl*/*kl*) mice, as shown in Figure 2. In WT but not in *kl*/*kl* mice, strong mKl-related staining was detected in distal nephron tubules, including DCT and CCD (Figure 2; Kl-Ab + Sec-Ab, Cortex; WT vs. *kl*/*kl*) and CNT. Weaker mKl-related staining was also detected in the BLM of PCT from WT but not *kl*/*kl* mice (Figure 2; Kl-Ab + Sec-Ab; Cortex; WT vs. *kl*/*kl*). Moreover, Sec-Ab gave no immunostaining when applied alone in WT kidneys (Figure 2; WT; Sec-Ab; CTX & OS). However, immunofluorescence staining was also observed in the brush border membrane (BBM) of PST S3 segments of both WT and *kl*/*kl* mice (Figure 2; Kl-Ab + Sec-Ab, Outer stripe; WT vs. *kl*/*kl*), thus indicating some non-specific mKl-related staining, whereas no visible mKl-related staining was detected in the BLM of S3 segments. Glomeruli of both WT and *kl*/*kl* mice (Figure 2; Kl-Ab + Sec-Ab; Cortex), as well as other nephron segments, including inner stripe and papilla, remained mKl-negative. In addition, no immunostaining was observed in the mKl-negative mouse livers. The Kl-antibody (AF1819) was also found useful in immunostaining cryosections of the rat kidneys, and was applied in further IFC studies in both mouse and rat kidneys.

### 2.2. Zonal Expression of rKl Along the Rat Nephron

**Figure 3:**
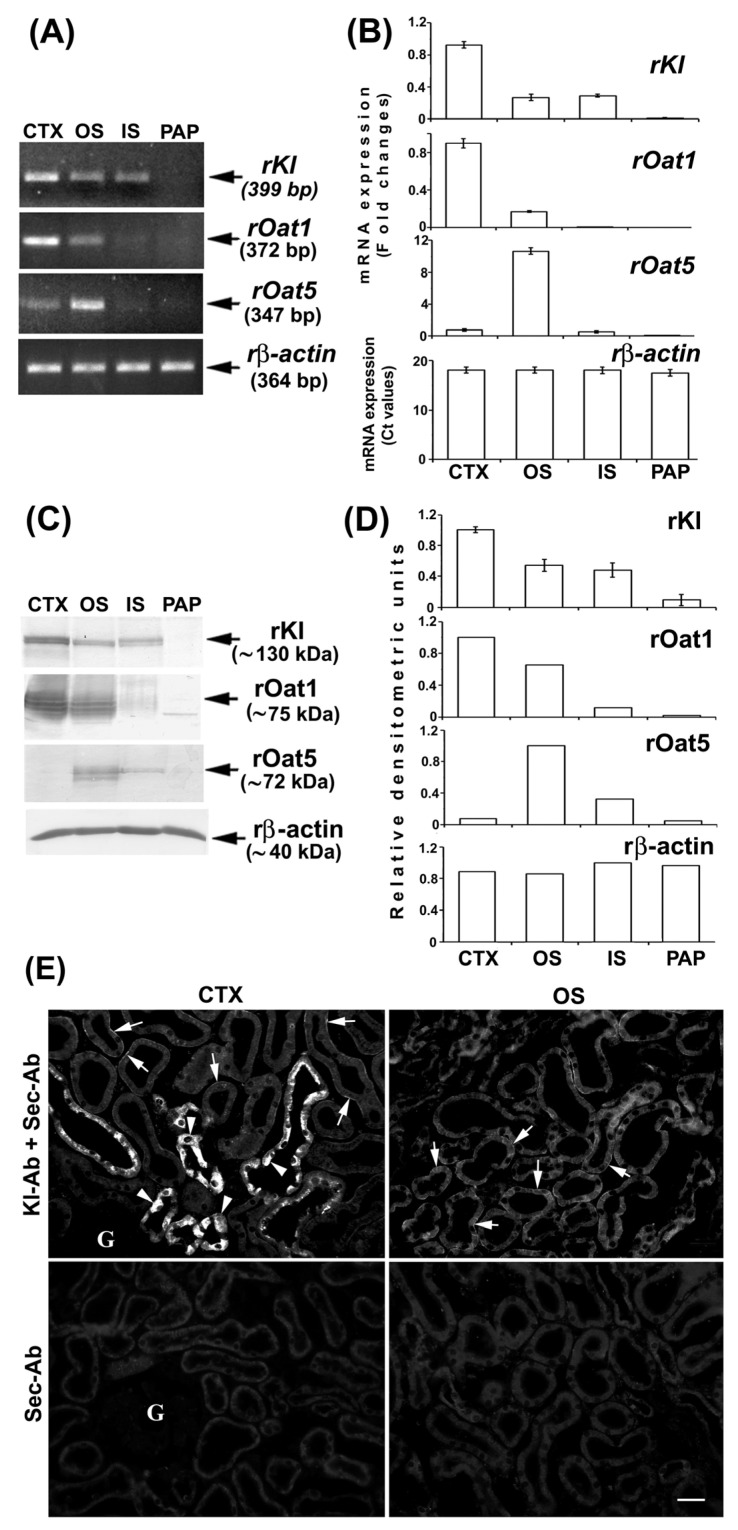
Expression and immunodistribution of rKl along the rat nephron. (**A**) Expression of *rKl*, *rOat1*, and *rOat5* mRNA in various nephron zones was studied by end-point RT-PCR using the housekeeping gene *rβ-actin* as an endogenous control. The figure shows images of agarose gel electrophoresis representative of similar findings in cDNA preparations from three male rats. (**B**) Expression of *rKl*, *rOat1*, and *rOat5* mRNA in various kidney zones was studied by qRT-PCR and analyzed by comparative 2^−ddCt^ method using the *rβ-actin* as endogenous control. The data are presented relative to the *rKl* mRNA in cortex; also shown is the mean ± SEM of data from cDNA preparations from three male rats. (**C**) Western blot of TCM proteins from various rat kidney zones was studied with Kl-Ab (KO603), Oat1-Ab, or Oat5-Ab using the rβ-actin as a loading control. Relative molecular mass (Mr, kDa) of protein bands was estimated by the protein ladder. Each well contained 80 μg protein. The figure also shows Western blot images representative of similar findings in TCM preparations from three male rats. (**D**) Densitometric evaluation of protein bands shown in C. Each bar is the mean ± SEM of data in TCM preparations from three male rats. (**E**) Immunodistribution of rKl protein in the rat kidneys was studied using the Kl-Ab (AF1819). The images of immunofluorescence staining representative of similar findings in cryosections from three male rats are also shown. Arrows, weak staining of the basolateral membrane of proximal convoluted tubules and proximal straight tubules in the cortex and outer stripe, respectively. Arrowheads, strong staining in epithelial cells of distal nephron segments. Individual tubules were identified based on their morphology and localization in specific nephron segments. The scale bar is 20 μm. CTX = cortex; G = glomerulus; IS = inner stripe; OS = outer stripe; Sec-Ab = secondary antibody; PAP = papilla.

We further investigated rKl distribution along the rat nephron at both mRNA and protein levels using the RT-PCR, WB, and IFC. Different zones of rat kidneys were manually dissected, and RNA and TCM from the cortex, outer stripe, inner stripe, and papilla were isolated. To check the quality and purity of the respective RNA and TCM samples, we initially tested the expression of rOat1/Slc22a6 and rOat5/Slc22a19 [34,35]. By RT-PCR, *rOat1* mRNA expression was highest in the cortex and much weaker in the outer stripe, whereas *rOat5* mRNA expression was highest in the outer stripe and much weaker in the cortex, while their mRNA level was negligible in the inner stripe and papilla (Figure 3A,B). The protein expression of these transporters (rOat1, ~75 kDa, and rOat5, ~71 kDa) followed a similar pattern (Figure 3C,D). The expression of rβ-actin was similar in all of the tested samples, indicating thus equal cDNA (Figure 3A,B) and protein (Figure 3C,D) loading. These findings resemble previously published data for rat kidneys [34,35]. Taken together, RNA and TCM samples from various rat nephron zones were not mutually contaminated, thus representing good quality samples for further rKl/rKl analysis at the mRNA and protein level, respectively. As shown in Figure 3A, the rKl-related RT-PCR product of 399 bp was highest in the cortex, similarly low in the outer and inner stripe, and negligible in the papilla. The same expression pattern was confirmed for the *rKl* mRNA by qRT-PCR (Figure 3B) as well as for the rKl-related protein band of ~130 kDa (Figure 3C,D). By IFC, using the Kl-Ab (AF1819), rKl-staining was detected in the cortex and outer stripe (Figure 3E), whereas no immunofluorescence was detected in the inner stripe and papilla. Similarly to mice, strong immunostaining was observed in distal nephron segments, including DCT, CNT, and CCD, whereas the BLM of PCT was stained more weakly (Figure 3E; Kl-Ab + Sec-Ab; CTX & OS). Furthermore, no fluorescence was detected in the cryosections of kidneys stained with the Sec-Ab alone (Figure 3E; Sec-Ab; CTX & OS), as well as in the *rKl-*negative liver stained with both primary and secondary antibodies. However, contrary to mice, rKl-related fluorescence was detected in BLM, but not BBM of PST in the outer stripe of the rat kidneys (Figure 3E; Kl-Ab + Sec-Ab; OS).

### 2.3. Cell-Specific Immunolocalization of Kl in Distal Segments of Rat and Mouse Nephron

**Figure 4:**
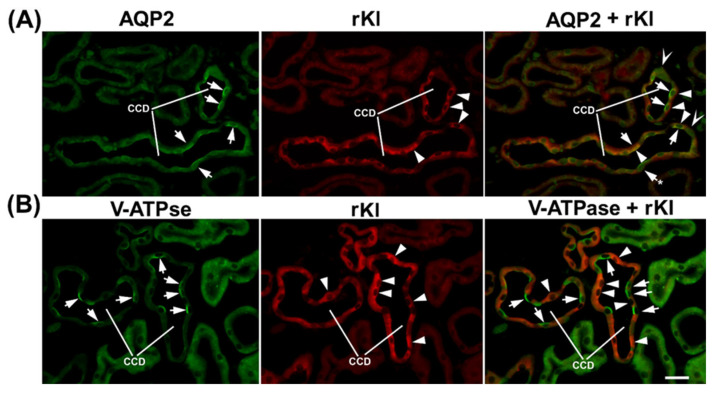
Cell-specific localization of rKl in the rat cortical collecting duct. Double-immunostaining was performed in the rat kidneys using the Kl-Ab (AF1819) with AQP2-Ab or V-ATPase-Ab. The figure shows images of immunofluorescence staining representative of similar findings in cryosections from three male rats. Cortical collecting ducts (CCD) were identified based on their localization and morphology. (**A**) AQP2 (green), rKl (red), and merged (AQP2 + rKl) images. Arrows—the AQP2-positive principal cells. Arrowheads—the rKl-positive cells. Arrows and Stars—the AQP2-positive principal cells without rKl-related staining. The cells labeled with Hollow Arrowheads are negative for both AQP2 and rKl. In most cases, AQP2 (arrows, green) and rKl (arrowheads, red) colocalized (arrows and arrowheads, merged) in the same cells, thus proving the presence of rKl in the principal cells of rat CCD. Occasional AQP2-positive principal cells stayed rKl-negative (arrow and star, merged). (**B**) V-ATPase (green), rKl (red), and merged (V-ATPase + rKl) images. Arrows—the V-ATPase-positive intercalated cells. Arrowheads—the rKl-positive cells. In rat CCD, V-ATPase (arrows, green) and rKl (arrowheads, red) did not colocalize (arrows and arrowheads, merged), thus proving the absence of rKl in V-ATPase-positive intercalated cells. The scale bar is 20 μm. CCD = cortical collecting ducts.

**Figure 5:**
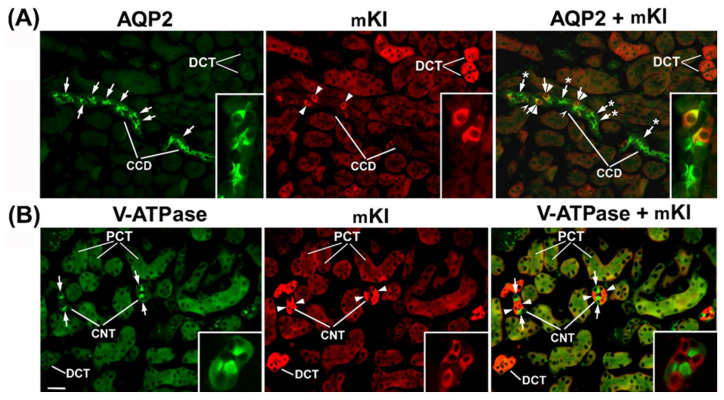
Cell-specific localization of mKl in the mouse cortical collecting ducts and connecting tubules. Double-immunostaining was performed in the cryosections of wild-type mouse kidneys using the Kl-Ab (AF1819) with AQP2-Ab or V-ATPase-Ab. Distal convoluted tubules (DCT), cortical collecting ducts (CCD), connecting tubules (CNT), and proximal convoluted tubules (PCT) were identified based on their localization and morphology. The figure shows images of immunofluorescence staining representative of similar findings in cryosections from three male wild-type mice. (**A**) AQP2 (green), mKl (red), and merged (AQP2 + mKl) images. Arrows—the AQP2-positive principal cells. Arrowheads—the mKl-positive cells. Arrows and Stars—the AQP2-positive principal cells without mKl-related staining. The cells labeled with Hollow Arrowheads are negative for both AQP2 and mKl (merged). The inset shows the CCD stained with AQP2-Ab and Kl-Ab in green, red, and merged mode. In some portion of mouse CCD, AQP2 (arrows, green) and mKl (arrowheads, red) colocalized (arrows and arrowheads, merged), thus proving the presence of mKl in the principal cells, whereas some AQP2-positive principal cells stayed mKl-negative (arrows and stars, merged). (**B**) V-ATPase (green), mKl (red), and merged (V-ATPase + mKl) images. Arrows—the V-ATPase-positive intercalated cells. Arrowheads—the mKl-positive epithelial cells. The inset shows the CNT stained with V-ATPase-Ab and Kl-Ab in green, red, and merged mode. In mouse CNT, V-ATPase (arrows, green) and rKl (arrowheads, red) did not colocalize (arrows and arrowheads, merged), thus proving the absence of mKl in the intercalated cells. The mKl-related staining is also visible in the basolateral membrane of proximal convoluted tubules (PCT). The scale bar is 20 μm for images in the same line, except for insets. CCD = cortical collecting ducts; CNT = connecting tubules; DCT = distal convoluted tubules; PCT = proximal convoluted tubules.

To define the Kl-positive cells in the CNT and CCD, we performed double-labeling IFC in the cryosections of rat and mouse kidneys in the same manner, since the distal portion between macula densa and CCD in rats was similarly organized to that in mice [19]. Double-staining was performed using the Kl-Ab (AF1819) and antibodies against water channel aquaporin-2 (AQP2-Ab) or 31 kDa subunit of vacuolar-type H+-ATPase (V-ATPase-Ab), as suitable markers for principal or intercalated cells, respectively [36,37]. In cortical distal tubules, the AQP2-Ab strongly stained the apical domain of principal cells in both rat (Figure 4A—green fluorescence) and mouse (Figure 5A—green fluorescence) kidneys. In the same sections, the Kl-Ab (AF1819) strongly stained the epithelial cells with reference to DCT and CCD (Figure 4A and Figure 5A—red fluorescence). The merged images clearly indicated a localization of Kl in AQP2-positive principal cells (Figure 4A and Figure 5A—merged). However, a portion of AQP2-positive cells remained Kl-negative (Figure 4A and Figure 5A—merged). In addition, the Kl-related fluorescence was not observed in the AQP2-positive principal cells residing in the outer and inner medullary CD of both rat and mouse kidneys. Furthermore, the V-ATPase-Ab stained the apical domain of intercalated cells in CNT and CCD from rat and mouse kidneys (Figure 4B and Figure 5B—green fluorescence), whereas in the same section, the Kl-Ab stained the adjacent V-ATPase-negative cells (Figure 4B and Figure 5B—red fluorescence). Thus, the merged images (V-ATPase + Kl) clearly indicated the absence of Kl in V-ATPase-positive intercalated cells in both species (Figure 4B and Figure 5B—merged). Also, in both species, Kl-related staining was not observed in V-ATPase-positive cells in the outer medullary and inner medullary CD. Taken together, these IFC results clearly revealed the localization of rKl and mKl in principal but not intercalated cells of cortical distal tubules in rodent kidneys.

### 2.4. Sex-Related Expression of Renal Kl in Rats and Mice

To study the sex-related expression of rKl in the rat kidneys, we employed an optimal experimental model described previously [35,38], which includes 3-month (mo)-old sham-operated and gonadectomised rats of both sexes. In these rats, we first measured the sex hormone concentrations in their blood plasma. As shown in Table 1, the plasma testosterone level was ~3.6-fold higher in sham-operated males than in sham-operated females, and ~91% lower in castrated than in sham-operated males. No significant change was found between sham-operated and ovariectomized females. In contrast, estradiol and progesterone plasma levels were respectively ~14-fold and ~2-fold higher in sham-operated females than in sham-operated males. While castration did not significantly change the levels of estradiol and progesterone in males, ovariectomy in females decreased the levels of these hormones by ~87% and ~51%, respectively.

**Figure 6:**
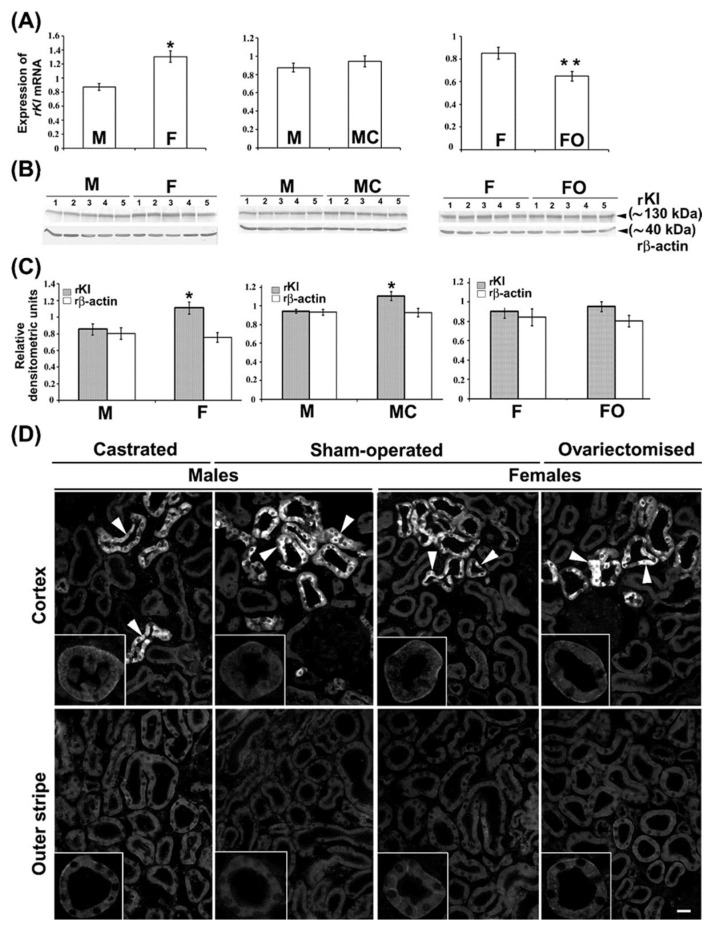
Female-dominant expression of rKl in the rat kidneys; effect of gonadectomy. (**A**) Expression of *rKl* mRNA in the kidneys of sham-operated and gonadectomised male and female rats was measured by qRT-PCR and analyzed by comparative 2^−ddCt^ method using the housekeeping gene *rβ-actin* as endogenous control. Data are presented relative to the *rKl* mRNA in the kidneys of sham-operated males or sham-operated females. Each bar is the mean ± SEM of the data in cDNA preparations from five rats in each experimental group. * *p* < 0.05, compared to sham-operated males. ** *p* < 0.05, compared to sham-operated females. (**B**) Expression of rKl protein in renal TCM of sham-operated and gonadectomised male and female rats was studied by Western blotting and Kl-Ab (KO603) using the rβ-actin as a loading control. Relative molecular mass (Mr, kDa) of protein bands was estimated by the protein ladder. Each well contained 80 μg protein. The figure shows images in TCM preparations from five rats in each experimental group. (**C**) Densitometric evaluation of protein bands shown in B. Each bar is the mean ± SEM of the data in TCM preparations from five rats in each experimental group. * *p* < 0.05, compared to sham-operated males. (**D**) Immunolocalization of rKl protein in the rat kidneys was studied by Kl-Ab (AF1819). The figure contains images of immunofluorescence staining representative of similar findings in cryosections from five rats in each experimental group. Arrowheads—the rKl-positive immunofluorescence staining in epithelial cells referring to distal tubules. Inset—the representative proximal convoluted tubules and proximal straight tubules in the cortex and outer stripe, respectively. The scale bar is 20 μm for all images except for the insets. F = sham-operated female; FO = ovariectomized female; M = sham-operated male; MC = castrated male.

We furthermore tested the expression of *rKl* mRNA and rKl protein in the kidneys of 3-month-old rats by qRT-PCR, WB, and IFC, as illustrated in Figure 6. Renal expression of *rKl* mRNA was ~50% stronger in sham-operated females as compared to sham-operated males (Figure 6A; M vs. F). The WB data matched the qRT-PCR data; abundance of the rKl-related protein band (~130 kDa) in renal TCM was approximately 40% stronger in sham-operated females than in males (Figure 6B,C; M vs. F), whereas expression of rβ-actin (~40 kDa) was similar in all TCM samples, demonstrating thus an equal protein loading (Figure 6B,C). In renal cryosections, rKl-related immunofluorescence in the BLM of both PCT and PST in cortex and outer stripe, respectively, was stronger in sham-operated females than males (Figure 6D; Cortex and Outer stripe; M vs. F; insets). In contrast to the subtle to moderate rKl-related staining in proximal tubules exhibiting a clear sex-related pattern (female > male), much stronger rKl-related staining in distal nephron segments (DCT, CNT, and CCD) was similar in sham-operated males and females (Figure 6D; Cortex; M vs. F). Collectively, these data indicated the presence of female-dominant expression of rKl in the rat kidneys, which was, however, visible only in the proximal tubule segments in the cortex and outer stripe (Figure 6A–D; M vs. F).

To examine the regulatory role of sex hormones on renal rKl expression, we tested the level of *rKl* mRNA and rKl protein in the kidneys of castrated males and ovariectomized females using the same analytical tools (qRT-PCR, WB, and IFC). Whereas castration had no effect (Figure 6A; M vs. MC), ovariectomy significantly downregulated (~24%) the expression of *rKl* mRNA when compared to sham-operated females (Figure 6A; F vs. FO). However, WB data in renal TCM exhibited a slightly different pattern; castration upregulated (~20%) the abundance of rKl protein band (Figure 6B,C; M vs. MC), while its expression in ovariectomized females remained unchanged (Figure 6B,C; F vs. FO). Expression of rβ-actin remained unaffected by gonadectomy of both males and females, demonstrating similar protein loading in all TCM samples (Figure 6B,C). Furthermore, IFC images showed similarity with WB data; castration increased the rKl-related immunofluorescence in the BLM of PCT and PST (Figure 6D; M vs. MC; Cortex and Outer stripe; insets), whereas ovariectomy had no effect (Figure 6D; F vs. FO; Cortex and Outer stripe; insets).

**Figure 7:**
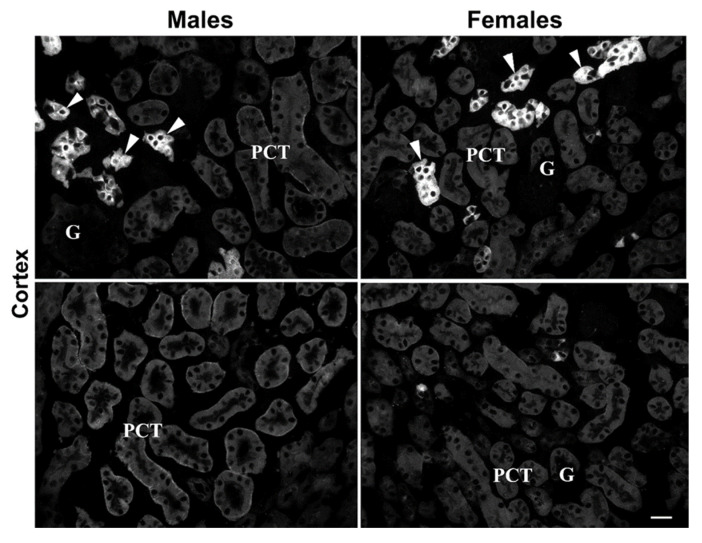
Male-dominant expression of mKl in the mouse kidneys. Immunodistribution of mKl in the mouse kidneys was studied by the Kl-Ab (AF1819). The figure shows images of immunofluorescence staining representative of similar findings in cryosections from four animals of each sex. Arrowheads—the mKl-positive staining in distal tubule cells. Different regions of cortex in the same animal of respective sex are also shown, having weaker (**upper panel**) or stronger (**lower panel**) mKl-fluorescence signals in BLM of proximal convoluted tubules. Scale bar is 20 μm. G = glomeruli; PCT = proximal convoluted tubules.

Possible sex differences regarding mKl-related staining in the mouse kidneys were tested by IFC in renal cryosections of 3-month-old male and female mice, as illustrated in Figure 7. Comparable to IFC findings in rats, strong mKl-related immunostaining was detected in the distal nephron segments of mouse kidneys, showing similar fluorescence intensity in both males and females (Figure 7: upper panel—Males vs. Females; arrowheads). However, mKl-related staining in BLM of PCT was heterogeneous, from barely to hardly visible staining scattered in some regions of the cortex (Figure 7: upper part of the panel) to stronger staining in other cortical regions (Figure 7: lower part of the panel). Contrary to IFC findings in rats, the overall intensity of mKl-related fluorescence in the BLM of PCT was stronger in male than female mice (Figure 7: upper and lower panel—Males vs. Females), indicating a possible presence of a male-dominant immunolocalization pattern of mKl in the mouse kidneys. This phenomenon was not further studied.

### 2.5. Ions (Pi and tCa) and Essential Macroelements (P and Ca) in Rat Plasma and Urine; Sex Differences

**Figure 8:**
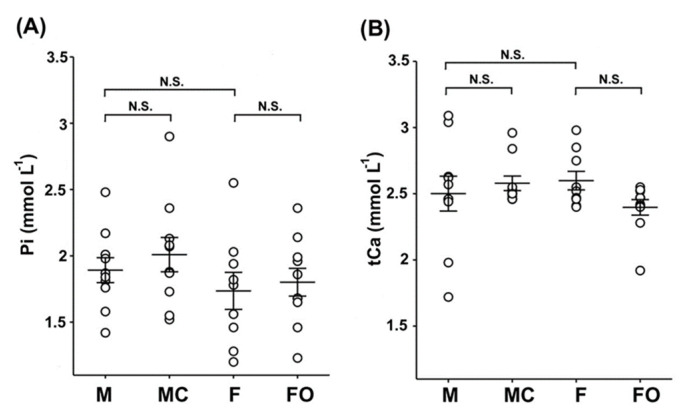
Inorganic phosphate (Pi) and total calcium (tCa) in rat plasma: sex differences and effect of gonadectomy. Concentrations of Pi (**A**) and tCa (**B**) were measured biochemically in the plasma of sham-operated and gonadectomised male and female rats. The figure shows data measured in plasma samples from 10 animals in each experimental group. Each dot represents a replicate of measured parameters; data are expressed as the mean ± SEM (*n* = 10). N.S. = no statistical significance at *p* < 0.05 evaluated by factorial ANOVA followed by Duncan post hoc test. F = sham-operated female; FO = ovariectomized female; M = sham-operated male; MC = castrated male.

Since Kl regulates mineral ion homeostasis by controlling the Pi and Ca^2+^ body concentrations, we assumed that the sex- and gonadectomy-related expression of renal rKl may influence the levels of these ions in rat plasma and urine. As shown in Figure 8, plasma concentrations of Pi (Figure 8A; M vs. F) and tCa (Figure 8B; M vs. F) were similar in sham-operated males and females, and remained unaffected by the gonadectomy (Figure 8A,B: M vs. MC and F vs. FO).

**Figure 9:**
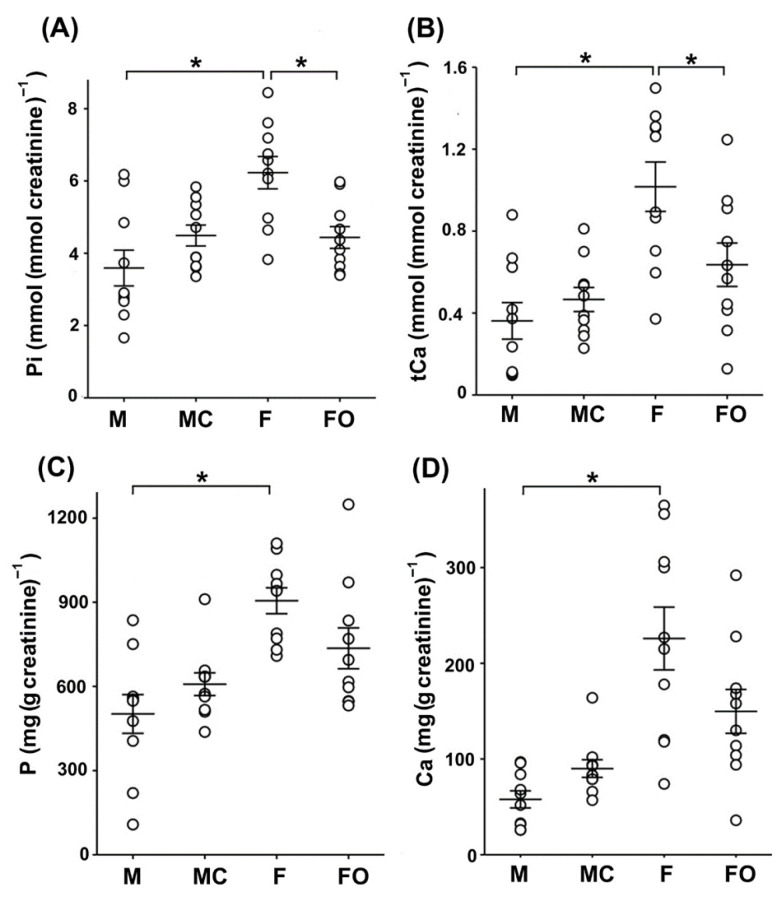
Inorganic phosphate (Pi), total calcium (tCa), phosphorus (P), and calcium (Ca) in rat urine. Urine samples were collected from sham-operated and gonadectomised male and female rats housed in metabolic cages for 24 h. The figure shows data measured in urine samples from 10 animals in each experimental group. Each dot represents a replicate of measured parameters; data are expressed as the mean ± SEM (*n* = 10). * Statistical significance at *p* < 0.05 was evaluated by factorial ANOVA followed by Duncan post hoc test. (**A**,**B**) Concentrations of Pi and tCa in urine samples were measured biochemically and normalized by urine creatinine concentrations. (**C**,**D**) Essential macroelements P and Ca in urine samples were measured by ICP-MS. The total content of P and Ca in urine samples was normalized to urine creatinine concentration. F = sham-operated female; FO = ovariectomized female; M = sham-operated male; MC = castrated male.

In urine, however, the level of Pi was ~70% higher in sham-operated females compared to sham-operated males (Figure 9A: M vs. F), whereas the urinary level of tCa was 180% higher in sham-operated females than in sham-operated males (Figure 9B: M vs. F). Furthermore, urinary concentrations of Pi and tCa were unaffected following castration in males (Figure 9A,B: M vs. MC), whereas in gonadectomised females, urinary concentrations of Pi and tCa were significantly lower (~29% and ~37%, respectively) compared to sham-operated females (Figure 9A,B: F vs. FO).

Using ICP-MS, we additionally measured the total content of the essential macroelements phosphorus (P) and calcium (Ca) in urine samples from sham-operated and gonadectomised rats (Figure 9C,D). The total content of P and Ca in urine was ~1.8-fold (for P) and ~3.9-fold (for Ca) higher in sham-operated females than in sham-operated males (Figure 9C,D: M vs. F), additionally demonstrating the sex-related pattern (females > males). Although the total content of P and Ca in rat urine was higher in castrated than in sham-operated males (Figure 9C,D: M vs. MC), and was lower in ovariectomized than in sham-operated females (Figure 9C,D: F vs. FO), their level was not statistically affected by castration and ovariectomy, respectively.

## 3. Discussion

We applied strategies for Ab validation using the relevant knockdown model as well as positive and negative controls according to well-known guidelines [39,40,41,42,43,44]. Our RT-PCR and WB data showed that commercial Kl-Ab (KO603), routinely used in mouse and human studies [5,6,17,45,46], could also be used for specific detection of the ~130 kDa full-length rKl^TM^ in TCM from *rKl*-positive kidney but not from *rKl-*negative liver [4,33]. Additional lower molecular weight bands observed in both kidney and liver were either not detected in the membrane fractions tested so far or not displayed on the cropped WB membranes [5,17,45,46]. The rKl-related band of ~70 kDa could represent a degradation product of rKl^TM^ in renal TCM (the most likely) or a cleaved portion of rKl^ecto^ as a residual impurity in renal and hepatic TCM (less possible) [5,6,17,18,45,46]. Secreted rKl^ecto^ could be cleaved into shorter rKl1 and rKl2 fragments of ~65 kDa by proteolytic cleavage at its linker region [7,8], and thus could potentially contaminate the renal and hepatic TCM samples. In contrast to humans and mice, alternative spliced variants of *rKl* mRNA, coding for the shorter rKl protein, were not detected in rats [3,4,21]. Our inability to detect either entire or smaller fragments of Kl^ecto^ in plasma and urine could have been due to the lower sensitivity of Kl-Ab (KO603) to detect its moderate level by WB in extracellular fluids, in contrast to highly expressed Kl-positive tissues or Kl-transfected cells [33]. Non-rKl-related bands of ~52–55 kDa in renal and hepatic TCM could be due to the non-specific binding of Sec-Ab since Kl-Ab (KO603) has been raised in rats as the host species [5]. In addition, batch-to-batch Ab variability can cause labeling of extra bands [39,40]. Using the Kl-Ab (KO603) and IFC, we could not detect any Kl-related staining in renal sections, even though numerous antigen-retrieval techniques were applied to reveal the hidden epitopes as detailed elsewhere [31,32]. It could be possible that Kl-Ab (KO603), generating against the synthetic peptide, may recognize a specific epitope of fully SDS-denatured but not native Kl in TCM and cryosections, respectively [5,39,41]. Our WB and IFC data thus collectively correlate with findings that commercial Kl-Ab (KO603) is applicable for WB, whereas its efficacy in immunocytochemistry varies between laboratories [5,33,47]. In contrast, we showed here that commercial Kl-Ab (AF1819) is applicable for Kl detection by IFC as previously reported [35]. Using the *Kl-*hypomorphic model, the Kl-Ab (AF1819) specifically stained the BLM and/or cytoplasm of various tubules in the cortex of WT mice with weak (PCT) or strong (DCT, CNT, and CCD) intensity and no signal in the outer and inner stripe as well as papilla, thus being in good agreement with several reports [1,5,6,14,15,16,17,33,45]. Although Kl^TM^ is a type I transmembrane protein anchored in the cell membrane, the strong cytoplasmic staining detected in distal cortical tubules corresponded highly with the cytoplasmic overlapping of Kl and Golgi as well as the endoplasmic reticulum [45,48]. In our hands, the Kl-Ab (AF1819) non-specifically stained the BBM of S3 segments in the mouse kidneys; the reason for this discrepancy was unclear since *mKl* mRNA was exclusively detected in the proximal convoluted (S1/S2 segments) but not proximal straight (S3 segments) tubules of the mouse cortex and outer stripe, respectively [6]. This could have occurred due to several aspects of the IFC procedure, including batch-to-batch Ab variations as well as applied antigen-retrieval methods [31,32,40,42,43,44]. In addition, it should be considered that mKl was previously detected in both BLM and BBM of PT by more sensitive immunogold electron microscopy [6].

Furthermore, we demonstrated the heterogeneous distribution of rKl in various segments of the rat nephron, where the quality of respective RNA and TCM samples was verified, demonstrating thus the cortex- and outer stripe-dominant expression of rOat1/Slc22a6 and rOat5/Slc22a19, respectively [34,35]. At both the mRNA and protein levels, rKl was cortex-dominant in comparison to its weaker level in the outer and inner stripe, and was negligible in the papilla. In line with RT-PCR and WB, rKl-staining was observed in cortical (DCT, CNT, CCD, and PCT) as well as outer stripe (PST) tubules without relevant immunostaining in the inner stripe and papilla, thus being in good agreement with Kato et al. [5]. The discrepancy between IFC and WB and qRT-PCR could have been due to lower rKl expression in the inner stripe, which therefore could not be detected by robust IFC, but was still detected by the more sensitive WB and the most sensitive RT-PCR. To the best of our knowledge, these comprehensive data, pointing out the zonal differences in rKl expression along the rat nephron [cortex >> (outer stripe > inner stripe)], have not been described so far. Although the expression and function of Kl in proximal and distal tubules have been extensively studied [2,6,9,11,12,13], all these findings raise questions about the potential role of rKl in the inner stripe. In the cell line of the mouse inner medullary collecting duct (mIMCD3), mKl was involved in the oxidative stress injury and apoptosis [49]. Despite the high sequence homology of *mKl* and *rKl* cDNA (93%) [1,4], the revealed species differences regarding the Kl distribution in the BLM of PST from rat but not mouse kidneys may collectively affect the outcome of further research.

To clarify the type of Kl-positive cells in the distal tubules of mouse and rat nephrons, we performed double-labeling by either AQP2-Ab or V-ATPase-Ab (31 kDa subunit), as suitable markers for principal and intercalated cells, respectively [36,37,50]. We demonstrated here that mKl and rKl were immunolocalized in the AQP2-positive principal cells located in CNT and cortical CD but not throughout the downstream outer and inner medullary CD, whereas intercalated cells with an apical V-ATPase (A-type) remained Kl-negative along both the mouse and the rat nephron. Although principal cells undoubtedly expressed Kl, some portion of AQP2-positive principal cells did not express Kl, both in mouse and rat kidneys. Since different cells along the distal convolution of rodent kidneys progressively replaced one another, creating thus a gradual segmental transition [18,19,20], it could be possible that some Kl-negative principal cells in cortical distal tubules belong to downstream CD principal cells with no mKl and rKl expression. Different subtypes of cells lining the distal tubules in mouse and rat nephrons mutually replace each other and intermingle as well [18,19,20], thus serving as one more plausible explanation for Kl’s unequal distribution in AQP2-positive principal cells. To the best of our knowledge, we are the first to describe in detail mKl and rKl localization in principal but no intercalating cells along distal nephron segments of both mouse and rat kidneys. At present, a possible correlation of these IFC findings with Kl function cannot be envisaged, although an expression/function of Kl altogether with various membrane proteins has been extensively studied in principal cells [48,51,52,53,54,55,56]. Overall, these nephron segments are critical for hormonal and non-hormonal regulation of multiple transporters/channels handling not only the ion transport, but also the water traffic; principal cells balance the electrolyte and fluid homeostasis [20,54,55,56,57], whereas intercalating cells regulate the acid-base homeostasis [37,50,55,58].

It should be emphasized that AQP2-positive principal cells in CD play a central role in the maintenance of body water homeostasis [56,57,59,60]. Klotho, highly expressed in distal tubular segments, including CCD, can influence the surface expression and stability of multiple renal transport proteins by modulating their glycosylation status, endocytic recycling, and proteolytic processing [61,62,63]. These mechanisms suggest that Kl may similarly modulate AQP2 membrane stability, trafficking, and post-translational regulation, thereby fine-tuning vasopressin-dependent water transport in CD. Further experimental studies are required to validate the proposed functional interaction between Kl and AQP2 in principal cells of CD to determine how this “AQP2–Kl axis” influences water reabsorption and tubular integrity under physiological and pathological conditions.

To demonstrate potential sex-related differences as well as the regulatory role(s) of sex hormones on renal rKl expression, we generated an optimal research model and measured sex hormones in the blood plasma of sham-operated and gonadectomised rats, as detailed elsewhere [35,38]. Accordingly, the male-dominant testosterone was strongly reduced by castration and was not affected by an ovariectomy, whereas female-dominant estradiol and progesterone were markedly reduced by ovariectomy and were not affected by castration, demonstrating thus the efficient removal of gonads as the key source of rat plasma sex hormones [38,64]. Although low levels of testosterone, estradiol, and progesterone were detected in the plasma of both sexes, the circulating sex steroids were mostly of gonadal origin, while some portions, especially the progesterone one, could have been derived from extra-gonadal sites, as reported elsewhere [38,64,65].

Using the qRT-PCR, WB, and IFC, we showed a female-dominant expression of renal rKl at both mRNA and protein; castration significantly upregulated rKl protein with no discernible effect on its mRNA, whereas *rKl* mRNA was downregulated by ovariectomy without effects on its protein level. This discrepancy suggests the existence of possible post-transcriptional and post-translational modifications of rKl, as reported for numerous genes and proteins, including renal membrane transporters in rats and mice [25,26,66].

More precisely, sex hormones may influence microRNA expression or RNA-binding proteins that could suppress or enhance rKl translation without altering its mRNA level [67,68,69]. Further post-translational modifications, including glycosylation, ubiquitination, proteolytic cleavage, and ectodomain shedding could additionally determine the rKl protein expression independently of its mRNA abundance [7,12,70]. Collectively, these mechanisms provide a robust explanation for the specific mismatches observed between mRNA and protein expression of rKl in the kidneys of gonadectomized rats.

Overall, the female-dominant expression of rKl could be largely regulated by the inhibitory effect of androgens (testosterone), whereas estrogens and/or progesterone could have a marginally regulatory role on renal rKl transcription/translation. In NRK-52E cells, dihydrotestosterone upregulated the expression of Kl in a time- and dose-dependent manner [30], pointing thus to the stimulating role of androgens on Kl expression. A plausible explanation of these contradictions could be due to complex tissue- as well as cell-specific interactions *in vivo* that cannot be simulated *in vitro*. Moreover, experimental setup and/or methodological differences may also challenge comparison *in vitro* vs. *in vivo* studies, emphasizing thereby the importance of unbiased *in vivo*/*ex vivo* studies to clarify the impact of sex steroids on renal rKl expression. Distinct sex-related expression of mKl was observed in the mouse kidneys; male-dominant mKl-related staining in the BLM of proximal (PCT) but not distal (DCT, CNT, and CCD) tubules was detected in accordance with the upregulated effect of testosterone on renal mKl mRNA/protein expression [30]. In the kidneys of *aromatase*-knockout mice with estrogen deficiency, mKl mRNA/protein was upregulated while estradiol normalized its renal expression, pointing to mKl as the estrogen-responsive gene [71]. Although *mKl* and *rKl* were reported as androgen-responsive genes having androgen response elements in their promoter regions, the existence of sex-response elements for estrogens and progestins could also be possible in *mKl* and *rKl* promoters, as well as their 3′ untranslated regions or those far distant from their coding region [30]. Furthermore, sex steroid receptors in mammalian kidneys participate in the gene/protein regulation of multiple membrane transporters/channels [25,27,28,29,72], thus liganded by androgens (most likely), estrogens, and progestins, and could also regulate renal transcription of *mKl* and *rKl* in a sex-related manner. Ligand-independent activation of sex steroid receptors, as well as non-transcriptional mechanisms of signal transduction through the sex steroid receptors, should not be excluded either [73]. Although sex hormone receptors have been discovered in mammalian kidneys, their specific localization along the nephron is still a subject of research [25,74]. In mice, classic nuclear receptors for progesterone, estradiol, and testosterone, as well as membrane-bound receptors for progesterone and estradiol, were detected in both proximal (PCT and PST) and distal (CCD) tubules in both sexes [75]. In rats, estrogen receptors have been detected in the renal cortex and medulla, while androgen receptors have been detected in proximal (PCT and PST) and distal (CCD) nephron segments [76,77]. All this data collectively suggests that both proximal and distal tubule cells expressing the Kl may represent the target site for sex hormones in rodent kidneys of both sexes. Therefore, further research should be conducted to link the sex-related expression of Kl and sex hormone receptors along the nephron, indicating the sex hormone roles in the regulation of Kl transcription. The sex-related findings of renal Kl expression presented here clearly suggest the use of both males and females in further studies, which is in line with numerous data as well as the SABV phenome emphasizing the use of both sexes despite higher study cost, as well as the widely accepted ‘’3Rs’’ concept [22,23,24,25,26,78]. Moreover, the interspecific differences (rats vs. mice) of renal Kl expression shown here should not be excluded in the interpretation of aging phenomena because of the well-documented contribution of sex hormones to longevity [79,80,81].

Keeping in mind the regulatory role of Kl on mineral ion homeostasis, ion (Pi and tCa) and essential macroelement (P and Ca) concentrations were tested in the rat urine and/or plasma. In accordance with Wistar rat Clinical Laboratory Parameters (https://www.criver.com/products-services/, accessed on 1 January 2025), the plasma level of Pi and tCa ions was approximately the same in both sexes (females ≈ males), and was accordingly refractory to gonadectomy. In contrast, the urinary level of Pi and tCa ions demonstrated a sex-related pattern (female > male) as reported previously [82], and was affected by ovariectomy but not castration. The sex-related expression of renal rKl shown here may thus help obtain a better understanding of mineral homeostasis. The female-dominant immunocytochemical pattern of rKl in the BLM of PCT and PST may represent the physiological microenvironment contributing to the phosphate status. In the BLM of PCT, Kl^TM^ activates the FGF23 signal transduction through the Kl-FGF23-FGFR complex, reduces the abundance of NaPi-IIa/SLC34a1 and NaPi-IIc/SLC34a3 on the BBM of PCT, and therefore suppresses the phosphate reabsorption in PT, thus regulating the phosphate homeostasis [10,11,15,83,84]. A similar inhibitory effect was observed in the *Kl*-positive oocytes, where Kl^TM^ downregulates *in vitro* both renal NaPi-IIa/SLC34a1 and intestinal NaPi-IIb/SLC34a2 [85]. Using the same FGF23 signaling pathway, Kl**^TM^** suppresses calcitriol synthesis in PCT, thus regulating the homeostasis of both Pi and Ca [10,86,87,88]. Also, soluble Kl^ecto^ in the luminal proto-urine can, independently of FGF23, inhibit the NaPi-IIa and NaPi-IIc in the BBM of PT, thus allowing phosphate excretion [6,8,89]. Moreover, the ICP-MS-measured macroelement P in rat urine was female-dominant, but was not affected by the gonadectomy. Keeping in mind that phosphorus in mammalian intracellular/extracellular compartments exists basically in phosphate form but not elemental phosphorus [90], the macroelement P level does not have to correlate with its ion Pi level since ICP-MS-detected phosphorus may originate from various phosphate-containing bioactive molecules in rat urine. Considering Kl as the key regulator of membrane abundance/activity for numerous transporters in PT that represent the principal sites for Pi reabsorption [6,8,10,11,15,83,84,89], female-dominant rKl expression in PT could potentially promote the phosphaturia, thus contributing to higher Pi levels in female urine.

In contrast to proximal tubules, rKl-related fluorescence in distal tubules exhibited a non-sex difference. Although rKl-staining in distal nephron segments (DCT, CNT, and CCD), representing the principal sites for Ca^2+^ handling, showed a non-sex-related pattern, the urinary level of the tCa ion was female-dominant. The urinary level of the Ca macroelement was also female-dominant, but was not affected by the gonadectomy; the ICP-MS-measured Ca in rat urine correlated well with the urinary level of the tCa ion, representing a combo of three Ca fractions, including free hydrated divalent cations Ca^++^, protein-bound calcium, and calcium complexed with various anions [91]. It has been reported that soluble Kl in proto-urine regulates the transport activity as well as membrane abundance of various channels/transporters *via* endocytosis/exocytosis. By preventing the internalization, Kl activates the calcium channel transient receptor potential vanilloid type 5 (TRPV5) and type 6 (TRPV6) in the apical membrane of DCT and CNT, where it promotes calcium transport, thus enhancing Ca^2+^ reabsorption and regulating the calcium homeostasis [6,8,9,14,89]. Furthermore, Kl^TM^ activates the FGF23 signaling pathways in the BLM of DCT and increases the abundance of TRPV5, thus enhancing Ca^2+^ reabsorption [10,11,92]. However, even though kidneys play a key role in phosphate and calcium handling, mineral homeostasis is regulated by a complex interplay between the parathyroid gland, kidneys, bone, and intestines through the feedback loops controlled by several key regulators, including parathyroid hormone, calcitriol, FGF23, and Klotho [90,93,94]. The undetected sex-related pattern of rKl-staining in DCT, CNT, and CCD may have also been due to the strong Kl expression in these nephron segments, and could be potentially detected using an extreme dilution of Kl-Ab. Taken together, the female-dominant expression of renal rKl, affected by prevailing inhibitory effects of testicular androgen (testosterone) and negligible stimulatory effect of ovarian-related estradiol and progesterone, may contribute to the sex-related level of rat urine electrolytes, particularly phosphate. This phenomenon may thus allow for a longer lifespan of female rats since Kl expression, together with Pi body levels affects aging [2,95].

## 4. Materials and Methods

### 4.1. Experimental Animals

Experiments were performed on 2.5-mo- to 3-mo-old male and female Wistar rats (outbred strain CRL: WI(Han)) from the breeding colony at the Institute for Medical Research and Occupational Health (Zagreb, Croatia), and on 3-mo-old male and female C57/Bl/6 mice from the breeding colony at the Ruđer Bošković Institute (Zagreb, Croatia). All animals were housed in standard environmental conditions under controlled room temperature (20–24 °C), humidity (40–60%), and lighting (12 h:12 h light–dark cycle). Standard rodent chow (Mucedola, Settimo Milanese, Italy) and tap water were available *ad libitum*. Renal tissue of 8-week-old *Kl-*hypomorphic (*kl*/*kl*) and control age-matched wild-type (WT, *kl^+^*^/*+*^) mice (129S1/SvImJ) were kindly provided by the University of Alabama at Birmingham (Birmingham, AL, USA) [33].

### 4.2. Surgical Procedure

Rats were randomly divided into 4 experimental groups with 5 animals/group (sham-operated males, sham-operated females, castrated males, and ovariectomized females), and were housed in separate cages. The gonadectomy of rats has been described in detail previously [35,64]. Briefly stated, rats were gonadectomized at an age of 6 weeks; males were bilaterally castrated *via* scrotal route, whereas females were bilaterally ovariectomized by dorsal (lumbal) approach under general anesthesia: Narketan (80 mg kg^−1^ body weight (BW) i.p.) and Xylapan (12 mg kg^−1^ BW i.p.). The sham-operated (control) animals underwent the same procedure, except that their respective organs were not removed. Operated animals were left to recover for another 6 weeks before being sacrificed at an age of 3 months. We did not exclude any animals.

### 4.3. Chemicals and Antibodies

Narketan and Xylapan were purchased from Chassot AG (Bern, Switzerland) All other chemicals and reagents were analytical and/or of molecular biology grade, and were obtained from various suppliers such as Kemika (Zagreb, Croatia), Sigma-Aldrich (St. Louis, MO, USA), or Fisher Scientific (New Jersey, NJ, USA). The sources of specific chemicals, reagents, and kits for RNA isolation, RT-PCR, membrane preparations, SDS-PAGE, WB, tissue fixation, IFC, ICP-MS, and measurement of sex hormones, Pi, and tCa are indicated in the text related to these methods. The various commercial and non-commercial primary Abs used in this study are listed in Table 2. Commercially secondary Abs were coupled to fluorochrome or alkaline phosphatase and are listed in Table 3, and used for IFC or WB, respectively.

### 4.4. Urine, Blood, and Tissue Sampling

Sampling of rat urine and blood plasma was performed as described in our recent papers [38,64]. Briefly described, two days before sacrifice, rats were placed individually in metabolic cages without access to food, with free access to tap water. Urine was collected for 24 h and centrifuged at 2500× *g* for 15 min to obtain a clear urine supernatant that was subsequently stored at −20 °C. The next day, rats were anesthetized i.p. with Narketan (80 mg kg^−1^ BW) and Xylapan (12 mg kg^−1^ BW), with the blood collected from the jugular vein into the heparinized vacutainer (Becton Dickinson, Franklin Lakes, NJ, USA) and centrifuged in a refrigerated (4 °C) centrifuge at 1000× *g* for 10 min to remove cells. The resulting plasma samples were stored at −20 °C until further use. Finally, rats were bled out by cutting the large vessels; liver and kidneys were removed, rinsed in an ice-cold phosphate-buffered saline (PBS), and appropriately sampled according to the specific methods described in further sections. Following the removal, the largest liver lobe was cut on ice in 1 mm-thick slices, whereas the kidneys were decapsulated and then transversally sliced on ice. In some experiments, the whole kidney was manually dissected into separate tissue zones (cortex, outer stripe, inner stripe, and papilla). The animals were sacrificed between 9:00 and 11:00 a.m.

### 4.5. RNA Isolation and RT-PCR

A hepatic slice (60–80 mg of wet tissue) and a middle transversal slice of each kidney (60–80 mg wet tissue) were immediately submerged in RNAlater (Sigma-Aldrich) at 4 °C overnight, followed by storage at −20 °C until further use. In some experiments, the whole kidney was manually dissected into separate tissue zones (cortex, outer stripe, inner stripe, and papilla), and tissues were separately submerged in RNAlater as described above. Total RNA from corresponding tissue slices was isolated by Trizol (Sigma-Aldrich), and subsequently purified by RNeasy Mini Kit (Qiagen, Germantown, MD, USA) following the manufacturer’s recommendation. Its purity and concentration were quantified by spectrophotometer BioSpec Nano (Shimadzu, Kyoto, Japan) at 260 and 280 nm, whereas its integrity was verified by electrophoresis in EtBr-stained agarose gel under UV. A cDNA was synthesized by High-Capacity cDNA RT-Kit (Applied Biosystems, USA) according to the manufacturer’s instructions, and stored at −20 °C. End-point RT-PCR was performed in a volume of 20 μL using 10 ng of first-strand cDNA template and PCR components (specific primers, dNTP mix, PCR buffer, and AmpliTaq DNA polymerase) (Applied Biosystems, Foster City, CA, USA) under thermal cycling using the PCR thermal cycler-2700 (Applied Biosystems, USA) as described in detail previously [35,96]. Specific intron over-spanning primers for rKl (F: 5′-AACAATGGCTTCCCTCCTTT-3′ and R: 5′-CACCACTGGAGTGATGTTGG-3′) were designed using the public software ‘’Primer 3.0” (http://frodo.wi.mit.edu/primer3/, accessed on 1 January 2025). Specific intron over-spanning primers for *rOat1*, *rOat5,* and housekeeping gene *rβ-actin* have been described elsewhere [35,96]. The optimal PCR cycle number was empirically determined for every tested gene, and RT-PCR products were checked by electrophoresis in EtBr-stained agarose gel under UV. Quantitative RT-PCR was performed in a volume of 25 μL using 10 ng of first-strand cDNA template and qPCR components (20X TaqMan Gene Expression Assays and 2X TaqMan Universal PCR Master Mix) (ThermoFischer Scientific, Applied Biosystems, Carlsbad, CA, USA) under thermal cycling conditions in the 7500 Real-Time PCR System (Applied Biosystems) as described previously [35]. The used TaqMan Gene Expression Assay IDs were as follows: Rn00580123_m1, Rn00568143_m1, Rn00696699_m1, and Rn00667869_m1 for rKl, rOat1, rOat5, and rβ-actin, respectively (https://www.thermofisher.com). Expression of *rKl*, *rOat1*, and *rOat5* mRNA was accomplished by comparative Ct method using the Relative Quantification Study Software v 1.3.1. (Applied Biosystems). To standardize the cDNA input, *rβ-actin* was quantified as an endogenous control, and results were normalized to these values. Each sample was performed in duplicate; the mean fold changes ± SEM of the two replicates were calculated. The non-template control (NTC), where the cDNA was substituted with nuclease-free water, was included in each PCR run; no products/signals were detected in NTC, confirming the absence of contamination.

### 4.6. Total Cell Membranes Preparation and Western Blotting

The hepatic and renal tissues were homogenized, and TCM isolation from respective tissue homogenates was performed by differential centrifugation as described in detail previously [35,64]. Briefly, 10% homogenates were centrifuged at 6000× *g* for 15 min to remove cell debris, and the supernatant was further centrifuged at 48,000× *g* for 1 h using the refrigerated (4 °C) centrifuge RC-5C (Sorwall Instruments, Newtown, CT, USA). Preparation of TCM samples and subsequent SDS-PAGE/WB analysis have been described in detail [34,35]. Briefly, TCM samples were mixed with loading buffer containing β-mercaptoethanol (reducing conditions) and denatured at 65 °C for 15 min. Proteins were separated through 10% SDS-PAGE mini gels and electrophoretically wet-transferred to the Immobilon membrane (Millipore, Bedford, MA, USA). Following transfer and Coomassie Brilliant Blue staining, Immobilon was destained and blocked with blotto-buffer (5% skimmed milk powder in PBS-Triton) for 1 h at room temperature. Afterward, it was incubated overnight at 4 °C with primary Abs diluted in blotto-buffer containing Kl-Ab (KO603, 1:500), Oat1-Ab (1:1000), Oat5-Ab (1:500), or β-actin-Ab (1:1000). Following washing, Immobilon was incubated with appropriate Sec-Ab for 1 h at room temperature, washed with blotto-buffer, and protein bands were visualized by the AP-mediated reaction [34,35]. The relative molecular mass of the labeled protein bands was estimated using the unstained Protein Ladder (Thermo Fischer Scientific). The labeled protein bands were scanned and analyzed by the public software Image J (https://imagej.nih.gov/ij/(accessed on 1 January 2025)). The density of protein bands was expressed in arbitrary units relative to the strongest band (=1 arbitrary unit). The amount of protein per well was indicated in the related Figure legends.

### 4.7. Tissue Fixation and Immunofluorescence Cytochemistry

Rats were anesthetized and then perfused *in vivo via* the heart’s left ventricle with 4% w v-1 paraformaldehyde (PFA) [31,32,59], whereas mice were anesthetized and then fixed *in vivo via* transcardial perfusion with Tyrode’s solution and 4% PFA fixative [33]. Afterwards, fixed tissue was incubated in 30% sucrose for 24 h at 4 °C, embedded in the Tissue Freezing Medium (Leica Biosystems, Newcastle upon Tyne, UK), frozen/sectioned at −25 °C using the Leica 1850-CM cryostat (Leica instruments GmbH, Nussloch, Germany), and 4 μm-thick cryosections were collected on Superfrost/Plus microscope slides (Thermo Fischer Scientific, Portsmouth, NH, USA) [31,32,59]. To obtain an optimal immunostaining in renal cryosections, various antigen-retrieval techniques were applied to maximize Ab binding as described in detail previously [31,32]. Additionally, antigen-retrieval was performed using the autoclave at 121 °C and ~2 bar. The protocol that gave the strongest Kl-related immunofluorescence in kidney included several steps as follows: (1) rehydration in PBS for 15 min; (2) heating in 10 mM citrate buffer; pH 6 (4 cycles in microwave oven at 800 W; 5 min each); (3) incubation in 0.5% Triton X-100 for 15 min; (4) incubation in 2% Triton X-100 for 15 min. Kidney sections were then rinsed in PBS (4 × 5 min each), incubated for 30 min in 1% bovine serum albumin to block the non-specific binding, followed by incubation in Kl-Ab (AF1819) (diluted in PBS, 1:5–1:250) overnight at 4 °C. Afterwards, they were washed in PBS (4 × 5 min each), incubated in DAG-CY3 (in PBS, 1:400) at room temperature for 60 min, washed in PBS (4 × 5 min each) and finally mounted in fluorescence fading retardant Vectashield (Vector Laboratories Inc., Burlingame, CA, USA). Immunostaining was inspected by Opton III RS fluorescence microscope (Opton Feintechnik, Oberkochen, Germany), and recorded by software-guided Spot RT Slider camera (Diagnostic Instruments, Sterling Heights, MI, USA). The images were imported into Adobe Photoshop 6.0 for processing and labeling. To double-stain Kl with AQP2 or V-ATPase, cryosections were first stained for Kl as described above, and then incubated with PBS-diluted AQP2-Ab (1:1) or V-ATPase-Ab (1:20) overnight at 4 °C, washed, and incubated with PBS-diluted GAR-FITC (1:100) or DAC-FITC (1:50), respectively, for 1 h at room temperature. Images of CY3-related red and FITC-related green fluorescence were merged in silico using Adobe Photoshop 6.0 software.

### 4.8. Sex Hormone Analysis

Concentrations of sex hormones (testosterone, estradiol, and progesterone) in the plasma were measured by commercially available chemiluminescent immunoassays using the VITROS ECi Immunodiagnostic System (Ortho-Clinical Diagnostic, Pencoed, UK) according to the manufacturer’s instructions, as reported previously [38,64].

### 4.9. Measurement of Inorganic Phosphate (Pi), Total Calcium (tCa), and Creatinine

Concentrations of Pi and tCa in native plasma and urine samples were measured using the automated analyzer Cobas^®^ 6000 (Roche Diagnostics GmbH, Mannheim, Germany), whereas the creatinine concentration in urine samples was measured using the Roche Enzymatic Creatinine Assay (Roche Diagnostics GmbH) according to the manufacturer’s instructions. Levels of Pi and tCa in urine samples were normalized to urinary creatinine concentrations.

### 4.10. Determination of Essential Macroelements

Concentrations of essential macroelements (P and Ca) in urine samples were analyzed by ICP-MS using an Agilent 7500cx (Agilent Technologies, Tokyo, Japan) as detailed previously [64,97].

### 4.11. Statistical Analysis and Data Presentation

Numeric data were statistically evaluated by Statistica 10 software (StatSoft Inc. Tulsa, OK, USA). The data were analyzed by Student’s *t*-test for comparison of 2 experimental groups; the amount of rKl-related protein band in renal membrane fraction was evaluated by Student’s *t*-test. Analysis of variance (ANOVA) followed by Duncan post hoc was used for the comparison of more than 2 experimental groups; expression of renal *rKl* mRNA as well as plasma and urine levels of sex hormones (testosterone, estradiol, and progesterone), biochemical parameters (Pi and tCa) and essential macroelements (P and Ca) were evaluated by factorial ANOVA followed by Duncan post hoc test. The values were expressed as the means ± standard error of means (SEM). Differences between groups were considered statistically significant at *p* < 0.05. Figures and images of qRT-PCR, IFC, WB, and sex hormone analysis represent findings in RNA samples, tissue cryosections, membrane and plasma samples, respectively, from 3 to 5 animals in each experimental group. Figures and Tables of biochemical parameters (Pi, tCa, and creatinine) and essential macroelements (P and Ca) represent findings in urine and plasma samples from 9 to 10 animals in each experimental group.

## 5. Conclusions

In summary, the sex-related findings of renal Kl expression in the common animal model presented here should be kept in mind in further basic, preclinical, and clinical studies to avoid any generalization of research data from just one sex, since various phenomena detected in one sex cannot be linearly translated to the other according to the SAVB phenomenon. Our results also bring to light the species differences in terms of sex-related expression of renal Kl as well as its immunodistribution in rat and mouse kidneys. Thus, we point out here that the species differences between mice and rats, as frequently used animal models, could also have an impact on further testing the mechanisms of aging and longevity, as well as applying the novel anti-aging discoveries to humans in clinical practice.

## Figures and Tables

**Table 1:** Sex hormones in the rat plasma: sex differences and effect of gonadectomy.

	Testosterone (nmoL L^−1^)	Estradiol (pmol L^−1^)	Progesterone (pmol L^−1^)
Sham-operated males	5.06 ± 0.78	26.86 ± 1.38 **	47.42 ± 5.85 **
Sham-operated females	1.41 ± 0.08 *	381.78 ± 66.25	109.26 ± 11.20
Castrated males	0.46 ± 0.02 *	31.78 ± 1.03	38.68 ± 4.17
Ovariectomized females	0.48 ± 0.11	50.4 ± 4.19 **	53.72 ± 8.51 **

Note for Table 1. Concentrations of sex hormones were analyzed in the plasma of sham-operated and gonadectomised rats of both sexes. Statistical analysis was evaluated by factorial ANOVA followed by Duncan post hoc test. Shown are the means ± SEM (*n* = 5 in each group). * *p* < 0.05, compared to sham-operated males. ** *p* < 0.05, compared to sham-operated females.

**Table 2:** Primary antibodies used in this study.

Protein	Antibody (Ab)	Catalog No. (Company/Manufacturer)
AQP2	AQP2-Ab	N/A (Non-commercial) [36,37]
beta-actin	β-actin-Ab	sc-47778 (Santa Cruz Biotechnology, Dallas, TX, USA) [32,35]
Kl	Kl-Ab	AF1819 (R&D Systems, Minneapolis, MN, USA) [33]
Kl	Kl-Ab	KO603; cloneKM2076 (TransGenicInc., Fukuoka, Japan) [5]
Oat1/Slc22a6	Oat1-Ab	N/A (Non-commercial) [34]
Oat5/Slc22a19	Oat5-Ab	N/A (Non-commercial) [35]
Vacuolar H+-ATPase (31 kDa-subunit)	V-ATPase-Ab	N/A (Non-commercial) [32,37]

Abbreviations in Table 2. AQP2 = water channel aquaporin 2; Kl = Klotho; Oat1 = Organic anion transporter 1; Oat5 = Organic anion transporter 5.

**Table 3:** Secondary antibodies used in this study.

Antibody	Catalog No.	Company/Manufacturer
DAC-FITC	703-095-155	Jackson ImmunoResearch Laboratories Inc. (West Grove, PA, USA)
DAG-CY3	705-166-147	Jackson ImmunoResearch Laboratories Inc.
GAM-AP	115-055-003	Jackson ImmunoResearch Laboratories Inc.
GAR-AP	111-055-003	Jackson ImmunoResearch Laboratories Inc.
GAR-CY3	111-165-003	Jackson ImmunoResearch Laboratories Inc.
GAR-FITC	02-15-06	Kirkegaard & Perry Laboratories (Geithersburg, MD, USA)
goat anti-rat IgG-AP	sc-2021	Santa Cruz Biotechnology
goat anti-rat IgG-FITC	sc-2011	Santa Cruz Biotechnology
RAG-AP	305-056-003	Jackson ImmunoResearch Laboratories Inc.

Abbreviations in Table 3. DAC-FITC = donkey anti-chicken immunoglobulin (IgG) coupled to fluorescein-isothiocyanate (FITC); DAG-CY3 = donkey anti-goat IgG coupled to indocarbocyanine (CY3); GAM-AP = goat anti-mouse IgG coupled to alkaline phosphatase (AP); GAR-AP = goat anti-rabbit IgG coupled to AP; GAR-CY3 = goat anti-rabbit IgG coupled to CY3; GAR-FITC = goat anti-rabbit IgG coupled to FITC.

## Data Availability

The original contributions presented in this study are included in the article. Further inquiries can be directed to the corresponding author.

## Abbreviations

The following abbreviations are used in this manuscript:

Ab	Antibody
AQP2	Aquaporin-2
BBM	Brush border membrane
BLM	Basolateral membrane
CCD	Cortical collecting ducts
CD	Collecting ducts
CNT	Connecting tubules
CTX	Cortex
DAC-FITC	Donkey anti-chicken IgG coupled to fluorescein-isothiocyanate
DAG-CY3	Donkey anti-goat IgG coupled to indocarbocyanine
DCT	Distal convoluted tubules
FGF23	Fibroblast growth factor 23
G	Glomerulus
GAM-AP	Goat anti-mouse IgG coupled to alkaline phosphatase
GAR-AP	Goat anti-rabbit IgG coupled to alkaline phosphatase
GAR-CY3	Goat anti-rabbit IgG coupled to indocarbocyanine
GAR-FITC	Goat anti-rabbit IgG coupled to fluorescein-isothiocyanate
IFC	Immunofluorescence cytochemistry
IS	Inner stripe
Kid	Kidney
Kl	Klotho
Kl^ecto^	Soluble form of Kl
KL^TM^	Membrane-bound Kl
Liv	Liver
Oat1	Organic anion transporter 1
Oat5	Organic anion transporter 5
OS	Outer stripe
Sec-Ab	Secondary antibody
PAP	Papilla
PCT	Proximal convoluted tubules
PST	Proximal straight tubules
SABV	Sex as biological variable
tCa	Total calcium
TCM	Total cell membranes
V-ATPase	Vacuolar-type H+-ATPase
WB	Western blotting
WT	Wild-type
